# Residence in a Distressed County in Appalachia as a Risk Factor for Diabetes, Behavioral Risk Factor Surveillance System, 2006-2007

**Published:** 2010-08-15

**Authors:** Lawrence Barker, Richard Crespo, Molly Shrewsberry, Robert B. Gerzoff, Darrlyn Cornelius-Averhart, Sharon Denham

**Affiliations:** Division of Diabetes Translation, Centers for Disease Control and Prevention; Marshall University, Huntington, West Virginia; Marshall University, Huntington, West Virginia; Centers for Disease Control and Prevention, Atlanta, Georgia; Centers for Disease Control and Prevention, Atlanta, Georgia; Ohio University, Athens, Ohio

## Abstract

**Introduction:**

We compared the risk of diabetes for residents of Appalachian counties to that of residents of non-Appalachian counties after controlling for selected risk factors in states containing at least 1 Appalachian county.

**Methods:**

We combined Behavioral Risk Factor Surveillance System data from 2006 and 2007 and conducted a logistic regression analysis, with self-reported diabetes as the dependent variable. We considered county of residence (5 classifications for Appalachian counties, based on economic development, and 1 for non-Appalachian counties), age, sex, race/ethnicity, education, household income, smoking status, physical activity level, and obesity to be independent variables. The classification "distressed" refers to counties in the worst 10%, compared with the nation as a whole, in terms of 3-year unemployment rate, per capita income, and poverty.

**Results:**

Controlling for covariates, residents in distressed Appalachian counties had 33% higher odds (95% confidence interval, 1.10-1.60) of reporting diabetes than residents of non-Appalachian counties. We found no significant differences between other classifications of Appalachian counties and non-Appalachian counties.

**Conclusions:**

Residents of distressed Appalachian counties are at higher risk of diabetes than are residents of other counties. States with distressed Appalachian counties should implement culturally sensitive programs to prevent diabetes.

## Introduction

Appalachia is a 205,000-square-mile region of the United States that follows the Appalachian Mountains from southern New York to northern Mississippi (1). The region includes all of West Virginia and parts of Alabama, Georgia, Kentucky, Maryland, Mississippi, New York, North Carolina, Ohio, Pennsylvania, South Carolina, Tennessee, and Virginia. Appalachia consists of 420 counties (410 in 2006 and 2007, the years we gathered our data). It has a population of approximately 24 million people, 42% of whom live in rural areas, compared with 20% of the national population (1). Appalachia's population in 2000 was 88% non-Hispanic white, compared with approximately 70% for the rest of the United States (2).

Historically, the people of Appalachia did not exhibit the mobility that characterized much of the rest of the United States and often remained on their ancestral land. As a result, they became isolated from the mainstream and culturally distinct from the rest of the nation (3). Today, Appalachia has high rates of poverty, low rates of education, high rates of unemployment, an aging population, limited access to health care, high rates of cigarette smoking, and generally poor health status (4,5). Poverty and low education (6), cigarette smoking (7), and advancing age (8) are all positively associated with diabetes. We speculated that, among the many health issues facing Appalachia, the region would have a high prevalence of diabetes.

We examined the relationship between residence in Appalachian counties (stratified by Appalachian Regional Commission [ARC]-defined classification, based on level of economic development) and self-reported diagnosed diabetes. We controlled for selected factors associated with diabetes.

## Methods

Although some counties that the ARC considers to be part of Appalachia might not fit all commonly held perceptions of the region, we used the ARC's definition to avoid controversy over what counties constitute Appalachia. Overall, counties classified by ARC as "distressed" tend to be the mountainous and isolated counties that most people consider to be Appalachia.

### Data source

The Behavioral Risk Factor Surveillance System (BRFSS) is a state-based system of repeated cross-sectional health surveys. The BRFSS annually assesses key behavioral risk factors and chronic conditions in noninstitutionalized US adults aged 18 years or older. Participants were selected from civilian residents with telephones by using random-digit–dialing methods (9). We used data from the combined 2006 and 2007 BRFSS from all states that contained at least 1 county that the ARC considered part of Appalachia in 2007. Self-reported diabetes status was assessed with the question, "Have you ever been told by a doctor that you have diabetes?" Women who reported having diabetes only during pregnancy were not counted as having diabetes. Our data source did not let us distinguish between type 1 and type 2 diabetes. Physical activity was assessed with the question, "During the past month, other than your regular job, did you participate in any leisure-time physical activity?" Smoking status was determined with the question, "Have you smoked at least 100 cigarettes in your entire life?" We calculated body mass index (BMI) as self-reported weight in kilograms divided by self-reported height in meters squared and defined obesity as ≥30 kg/m^2^. Sociodemographic characteristics (age, race/ethnicity, sex, education, and income) were self-reported.

### Classification of counties

The ARC measures development of counties by comparing 3-year unemployment rate, per capita income, and poverty rate with corresponding national rates. The ARC classifies Appalachian counties as distressed (worst 10% compared with all counties in the nation), at risk (worst 10% to 25%), transitional (worst 25% to best 25%), competitive (best 25% to 10%), and attainment (best 10%) (Figure). County classification can change over time, but changes are often slow. We used the classification as of 2007.

**Figure.**
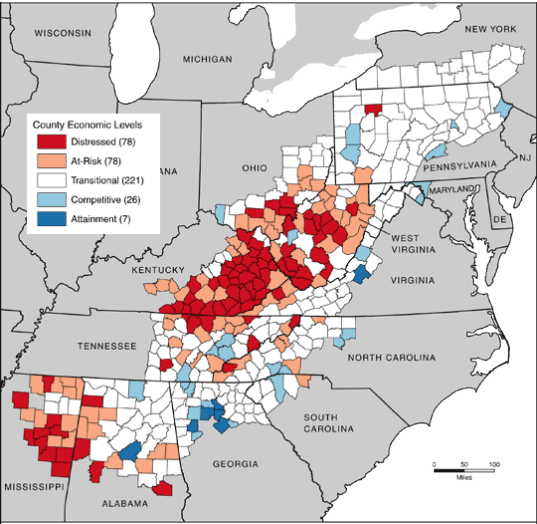
Map of Appalachia showing county development level, 2007. Source: Appalachian Regional Commission (ARC). The ARC uses an index-based county economic classification system to identify and monitor the economic status of Appalachian counties. Data sources: US Bureau of Labor Statistics, Local Area Unemployment Statistics, 2002-2004; US Bureau of Economic Analysis, Regional Economic Information System, 2003; US Census Bureau, 2000 Census, Summary File 3.

### Statistical analysis

We conducted a person-level analysis, treating classification of county of residence at the time of survey as an exposure. We used descriptive statistics to compare people by classification of county of residence. We conducted a logistic regression that used self-reported diagnosed diabetes as the dependent variable. The independent factors considered were classification of county of residence, age, sex, race/ethnicity, education, annual household income, cigarette smoking, physical activity, and obesity. To minimize the effect that variability among state policies and programs might have on our conclusions, we restricted our analyses to states that contain at least 1 Appalachian county. Thus, the term "non-Appalachian counties" refer to the non-Appalachian counties within the 13 Appalachian-associated states. We also compared distressed counties with other Appalachian counties.

To verify that the distressed counties differed from other Appalachian counties, we repeated the analysis using distressed Appalachian counties as the reference group. To examine the effect of dichotomizing BMI, we conducted a parallel analysis using BMI and BMI^2^ as continuous variables. We conducted weighted analyses using SAS version 9.2 (SAS Institute, Inc, Cary, North Carolina) to account for complex sample design. We considered results significant at *P* < .05.

## Results

BRFSS data indicate that the prevalence of diabetes is 10% for Appalachia and 8% nationally. Our data set consisted of 46,355 respondents from Appalachian counties and 150,679 respondents from non-Appalachian counties in states that contained some part of Appalachia.

The unadjusted prevalence of diabetes by county classification ranged from 6% for the attainment counties (95% confidence interval [CI], 5%-8%) to 13% for distressed counties (95% CI, 12%-15%), compared with the national median prevalence of 8% (10) (Table 1). The unadjusted prevalence of obesity and physical inactivity, risk factors for diabetes, is higher in the distressed counties than in other counties (Table 1). Unadjusted prevalence is calculated so that the magnitude of the problem of diabetes in Appalachia can be readily seen.

Controlling for covariates, residents of distressed counties in Appalachia had 33% higher odds of reporting diabetes than residents in non-Appalachian counties (*P* = .003) (Table 2). After accounting for other risk factors, we found no evidence that the risk of diabetes differed between non-Appalachian counties and Appalachian counties not classified as distressed.

In the analysis using distressed Appalachian counties as the reference group (data not shown), the odds ratios for Appalachian counties ranged from 0.75 (95% CI, 0.60-0.93) for competitive counties to 0.78 (95% CI, 0.62-0.98) for at-risk counties. Residents of counties of all classifications except attainment counties were significantly less likely to report diabetes than were residents of distressed counties.

In the analysis treating BMI as a continuous variable, the odds ratio for distressed counties, with non-Appalachian counties as reference, was 1.40 (95% CI, 1.10-1.80). Odds ratios for other county types ranged from 0.90 to 1.10 and were not significantly different from 1.00.

## Discussion

This study is, to our knowledge, the first quantitative assessment of diabetes in the Appalachian region as a whole. We found that Appalachians living in distressed counties are at higher risk of diabetes than are residents in other Appalachian counties.

Residents of nondistressed counties, with their higher incomes and levels of education, tend to be of higher socioeconomic status (SES) than do residents of distressed counties. Many factors, such as access to health care, social and cultural attitudes, direct effects of lower SES, and environmental factors, could contribute to the higher risk of diabetes in the distressed counties.

Sixty-nine percent of Appalachian counties and 91% of the distressed counties are designated as Health Professions Shortage Areas (11). This shortage could contribute to people seeking care late in the course of their diabetes or not getting preventive services to prevent or delay diabetes.

Access is only one side of the medical care equation. Social and cultural factors could also affect the amount and quality of care. Literacy and health literacy are low in the Appalachian population (Denham S, Rathbun A. Evaluating health literacy and information seeking behaviors in Appalachia. Unpublished manuscript). Appalachian participants in 2 focus group studies tended to have a low level of medical knowledge (12,13). Cultural barriers also are present. Appalachians are often reluctant to seek medical advice (12). Focus group participants stated that Appalachian people are belittled by some health care providers for their speech patterns and idioms (13). Similarly, some health care professionals have inadequate cultural competence (14). A large number of foreign-born physicians work in Appalachia, and their cultural differences can be a barrier to seeking care (13).

Several factors could contribute to the high rates of obesity and physical inactivity in Appalachia. Obesity is associated with low SES (15,16), and residents of distressed counties typically have low SES. Food insecurity (unreliable access to food) often affects low-SES people. Some studies have found an association between food insecurity and obesity (17-21).

Environmental factors in Appalachia could contribute to poor eating habits and physical inactivity, which are risk factors for obesity and diabetes. Full-service grocery stores are scarce, and residents of distressed counties often have easier access to convenience stores. One study of convenience stores in an Appalachian county found that no convenience store carried fresh or frozen green or yellow vegetables, low-fat milk or yogurt, or low-fat cheese (22). In another study conducted in Appalachia, adolescents reported eating "junk food" because healthy alternatives were unavailable (23). Others living in Appalachia have reported similar access issues as a barrier to eating a healthy diet (24).

The lack of access to environmental resources for physical activity is another factor that could contribute to diabetes prevalence. Distressed counties are usually rural, and residents may lack the time and money to reach facilities that are appropriate for physical activity (24,25). Additionally, few neighborhoods have streets and sidewalks where people can safely walk for exercise.

Programs aimed at lowering obesity and increasing physical activity in Appalachia, particularly in distressed counties, may lower the prevalence of diabetes. Two such programs are the Appalachian Diabetes Control and Translation Project and the *Diabetes: A Family* *Matter* program and tool kit. Neither has been formally evaluated.

The Appalachian Diabetes Control and Translation Project is a joint project of the Centers for Disease Control and Prevention, the ARC, and Marshall University's Robert C. Byrd Center for Rural Health. This project promotes community coalitions to involve the community, mobilize local resources, and develop local leadership (26). Since 2001, the project helped create 67 local coalitions that address diabetes and its complications in rural Appalachia through cooking classes, support groups, and walking clubs. Fifty-eight of the coalitions were still active at the time of our study, offering evidence that the coalitions are sustainable (27).

Unique cultural traits of Appalachian traditions should be considered in the development and use of health education materials (28). The *Diabetes: A Family* *Matter* program and tool kit were created with the recognition that the family and family-centered activities are important to rural Appalachian society. This program delivers culturally sensitive messages to increase awareness about diabetes risks, self-management, and healthy lifestyles. The program and associated tool kit encourage the participation of a local leader, preferably a diabetes educator or someone with expertise about diabetes. Citizen action, local coalitions, and volunteers are emphasized (29).

Our analysis is subject to several limitations. First, BRFSS data are self-reported and subject to nonreporting bias and social desirability bias. Similarly, BRFSS excludes households without land-line telephones, which introduces its own bias. Second, we could consider only diagnosed diabetes. Nationally, approximately 24% of type 2 diabetes cases are undiagnosed (30). The reluctance of people in Appalachia to seek medical advice could result in delayed diagnoses and a higher prevalence of undiagnosed diabetes than the national average. Third, we determined county of residence at the time of the survey. Diabetes is a chronic disease that often develops slowly. Respondents may have lived in a different county when they developed diabetes. Furthermore, ARC classifications of county development can change, and we used the classifications as of 2007. Finally, we studied prevalence, not incidence, of diabetes. Because the incidence of diabetes in a given period is much lower than the prevalence, incidence is harder to study. However, the primary determinants of diabetes prevalence are cumulative incidence and death rate. To show that people in distressed counties are at higher risk, we believe that study of prevalence is sufficient.

Residents of distressed Appalachian counties are at substantial risk of diabetes. Age, race, and sex are not modifiable; education (except possibly for the young) and income are difficult to modify. Physical activity, smoking, and obesity are all modifiable, and thus should be the focus of interventions intended to prevent diabetes. Although the direction of causality between smoking and diabetes is not clearly established, the direction for physical activity and obesity is clear: obesity and lack of physical activity directly contribute to diabetes. Obesity and lack of physical activity, which are common in distressed Appalachian counties, contribute to, but do not completely account for, this risk. We recommend that residents of distressed Appalachian counties be considered a health disparity population. Furthermore, we recommend that states containing Appalachian counties, particularly the distressed counties, consider implementing culturally sensitive programs, preferably using community members. These programs should promote physical activity and increase understanding of physical activity as a means of weight loss.

Finally, our findings do not mean that residents of nondistressed Appalachian counties are not at an elevated risk of diabetes. Some nondistressed Appalachian counties have high rates of obesity and lack of physical activity as well as other risk factors for diabetes. Policy makers and providers should consider all these factors when determining which counties are in most need of efforts to prevent diabetes.

## Figures and Tables

**Table 1:** Characteristics of Respondents (N = 197,034) by County of Residence, Appalachian Region, Behavioral Risk Factor Surveillance System, 2006-2007

Characteristic	ARC County of Residence^a^						Overall, % (95% CI)n = 197,034

	Distressed, % (95% CI) n = 2,608	At-risk, % (95% CI) n = 7,636	Trans, % (95% CI) n = 27,269	Comp, % (95% CI) n = 7,152	Attain, % (95% CI) n = 1,690	Non-App, % (95% CI) n = 150,679	
**Self-reported diabetes^b^**	13 (12-15)	11 (10-12)	10 (9-11)	9 (8-10)	6 (5-8)	9 (8-9)	9 (9-9)
**Age, y**							
≤44	50 (47-53)	48 (45-51)	47 (44-50)	49 (45-54)	62 (58-66)	51 (46-53)	51 (40-53)
45-64	34 (32-36)	35 (33-37)	34 (33-36)	33 (31-35)	30 (26-32)	33 (32-34)	33 (32-34)
≥65	16 (14-18)	17 (15-19)	19 (17-21)	18 (15-21)	8 (7-10)	16 (14-17)	16 (15-17)
**Sex**							
Men	47 (44-50)	47 (46-49)	47 (46-49)	49 (47-50)	50 (48-53)	48 (48-49)	48 (48-48)
Women	53 (50-56)	53 (51-54)	53 (51-54)	51 (50-53)	50 (47-52)	52 (51-52)	52 (52-52)
**Annual income, $**							
≥50,000	27 (24-31)	28 (26-31)	39 (37-41)	47 (44-50)	66 (63-70)	48 (46-50)	47 (45-48)
35,000 to <50,000	16 (14-18)	19 (17-21)	18 (17-19)	17 (16-18)	13 (12-16)	16 (15-16)	16 (15-17)
25,000 to <35,000	14 (12-16)	14 (13-16)	13 (12-14)	12 (11-14)	8 (6-9)	12 (12-12)	12 (12-12)
15,000 to <25,000	23 (20-25)	23 (21-25)	19 (18-20)	15 (14-17)	9 (6-12)	16 (15-16)	16 (15-17)
<15,000	21 (18-23)	15 (13-18)	11 (10-12)	9 (8-10)	4 (3-6)	9 (8-10)	9 (9-10)
**Race/ethnicity**							
Non-Hispanic white	89 (82-93)	86 (80-92)	86 (82-89)	78 (73-83)	68 (61-74)	70 (67-74)	72 (69-76)
Non-Hispanic black	8 (4-16)	9 (5-16)	8 (5-12)	13 (9-18)	14 (11-18)	16 (14-19)	15 (13-18)
Hispanic or Latino	1 (1-2)	2 (1-2)	3 (2-4)	4 (3-5)	9 (7-12)	7 (5-10)	6 (5-9)
Non-Hispanic multiracial	1 (0-1)	1 (1-2)	1 (1-1)	1 (1-2)	1 (1-2)	1 (1-2)	1 (1-1)
Non-Hispanic other	1 (1-1)	2 (1-2)	2 (2-3)	4 (3-5)	8 (6-11)	5 (4-6)	4 (4-6)
**Education**							
College or technical school graduate	16 (13-18)	19 (17-21)	26 (25-28)	35 (31-39)	48 (44-51)	33 (31-35)	32 (31-34)
Some college or technical school	23 (20-26)	24 (22-26)	25 (24-26)	26 (24-28)	25 (22-28)	25 (24-26)	25 (25-26)
High school graduate	38 (35-41)	40 (37-43)	36 (34-38)	30 (27-32)	22 (19-26)	31 (29-33)	31 (30-33)
Less than high school graduate	24 (21-26)	18 (16-20)	12 (11-14)	9 (7-11)	6 (4-7)	11 (10-12)	11 (10-12)
**Smoking^b^**							
No	47 (44-51)	48 (46-51)	52 (51-54)	54 (52-55)	62 (58-66)	55 (54-56)	55 (54-55)
Yes	53 (49-56)	52 (49-54)	47 (46-49)	47 (45-48)	38 (34-42)	45 (44-46)	45 (45-46)
**Physical activity^b^**							
No	37 (34-40)	32 (30-35)	26 (25-28)	22 (21-23)	20 (18-22)	25 (24-26)	25 (24-26)
Yes	63 (60-66)	68 (65-70)	74 (72-75)	78 (77-79)	80 (78-82)	75 (74-76)	75 (74-76)
**Obesity^b^**							
No	63 (61-66)	70 (68-73)	71 (70-72)	74 (73-76)	76 (74-78)	73 (72-74)	73 (72-74)
Yes	37 (34-40)	30 (27-32)	29 (28-30)	26 (24-27)	24 (22-26)	27 (26-28)	27 (26-28)

Abbreviations: ARC, Appalachian Regional Commission; CI, confidence interval; Trans, Transitional; Comp, Competitive; Attain, Attainment; Non-App, Non-Appalachian.

a The ARC compares 3-year unemployment rate, per capita income, and poverty rate with corresponding national rates and classifies Appalachian counties as follows: distressed (worst 10% compared with all counties in the nation), at-risk (worst 10% to 25%), transitional (worst 25% to best 25%), competitive (best 25% to 10%), and attainment (best 10%).

b See Methods for definition.

**Table 2:** Odds of Self-Reported Diabetes^a^ Among Respondents (N = 197,034^b^), Appalachian Region, Behavioral Risk Factor Surveillance System, 2006-2007

**Characteristic**	AOR (95% CI)	*P* Value^c^
**ARC county of residence^d^**		
Non-Appalachian	1 [Reference]	NA
Distressed	1.33 (1.10-1.60)	.003
At-risk	1.04 (0.90-1.18)	.54
Transitional	1.05 (0.97-1.14)	.24
Competitive	1.00 (0.88-1.13)	.96
Attainment	1.00 (0.78-1.28)	.99
**Age, y**		
≤44	1 [Reference]	NA
45-64	4.86 (4.44-5.31)	<.001
≥65	9.10 (8.32-9.96)	<.001
**Sex**		
Men	1 [Reference]	NA
Women	0.71 (0.68-0.76)	<.001
**Annual income, $**		
≥50,000	1 [Reference]	NA
35,000 to 50,000	1.26 (1.15-1.38)	<.001
25,000 to <35,000	1.35 (1.22-1.48)	<.001
15,000 to <25,000	1.59 (1.45-1.74)	<.001
<15,000	2.09 (1.89-2.31)	<.001
**Race/ethnicity**		
Non-Hispanic white	1 [Reference]	NA
Non-Hispanic black	1.63 (1.50-1.76)	<.001
Hispanic or Latino	0.99 (0.82-1.20)	.94
Non-Hispanic multiracial	1.36 (1.07-1.72)	.01
Non-Hispanic other	1.45 (1.17-1.79)	<.001
**Education**		
College or technical school graduate	1 [Reference]	NA
Some college	1.20 (1.07-1.32)	<.001
High school graduate	1.15 (1.06-1.25)	<.001
Less than high school graduate	1.19 (1.07-1.33)	.002
**Cigarette smoking^a^**		
No	1 [Reference]	NA
Yes	1.10 (1.04-1.17)	.003
**Physical activity^a^**		
Yes	1 [Reference]	NA
No	1.37 (1.29-1.45)	<.001
**Obesity^a^**		
No	1 [Reference]	NA
Yes	3.29 (3.11-3.48)	<.001

Abbreviations: AOR, adjusted odds ratio; CI, confidence interval; ARC, Appalachian Regional Commission; NA, not applicable.

a See Methods for definition.

b Values for 38,552 respondents were excluded because of missing/don't know responses.

c Calculated by using the Wald test.

d The ARC compares 3-year unemployment rate, per capita income, and poverty rate with corresponding national rates and classifies Appalachian counties as follows: distressed (worst 10% compared with all counties in the nation), at-risk (worst 10% to 25%), transitional (worst 25% to best 25%), competitive (best 25% to 10%), and attainment (best 10%).
